# The optimal PEEP after alveolar recruitment maneuver assessed by electrical impedance tomography in healthy horses

**DOI:** 10.3389/fvets.2022.1024088

**Published:** 2022-12-09

**Authors:** Felipe Silveira Rego Monteiro Andrade, Aline Magalhães Ambrósio, Renata Ramos Rodrigues, Lara Lopes Faccó, Lucas Alaião Gonçalves, Sérgio Grandisoli Garcia Filho, Rosana Thurler dos Santos, Thais Colombo Rossetto, Marco Aurélio Amador Pereira, Denise Tabacchi Fantoni

**Affiliations:** Department of Surgery, School of Veterinary Medicine and Animal Science, University of São Paulo, São Paulo, SP, Brazil

**Keywords:** atelectasis, oxygenation, pulmonary ventilation, electrical impedance tomography, horses, alveolar recruitment maneuver, PEEP, overdistention

## Abstract

**Background:**

Electrical impedance tomography (EIT) has been an essential tool for assessing pulmonary ventilation in several situations, such as the alveolar recruitment maneuver (ARM) in PEEP titration to maintain the lungs open after atelectasis reversion. In the same way as in humans and dogs, in horses, this tool has been widely used to assess pulmonary aeration undergoing anesthesia, mechanical ventilation, recruitment maneuver, standing horses, or specific procedures.

**Objectives:**

The present study aimed to evaluate the distribution of regional ventilation during ARM based on lung monitoring assessment by EIT, with a focus on better recruitment associated with less or no overdistention.

**Methods:**

Fourteen horses of 306 ± 21 kg undergoing isoflurane anesthesia in dorsal recumbency were used. The animals were mechanically ventilated with a tidal volume of 14 ml kg^−1^ and a respiratory rate of 7–9. An alveolar recruitment maneuver was instituted, increasing the PEEP by five cmH_2_O every 5 min until 32 cmH_2_O and decreasing it by five cmH_2_O every 5 min to 7 cmH_2_O. At each step of PEEP, arterial blood samples were collected for blood gas analysis, EIT images, hemodynamic, and respiratory mechanics.

**Results:**

Associated with the CoV-DV increase, there was a significant decrease in the DSS during the ARM and a significant increase in the NSS when PEEP was applied above 12 cmH_2_O compared to baseline. The Compl_ROI_ showed a significant increase in the dependent area and a significant decrease in the non-dependent area during ARM, and both were compared to their baseline values. The driving pressure decreased significantly during the ARM, and Cst, PaO_2_, and PaO_2_/FiO_2_ ratio increased significantly. The V_D_/V_T_ decreased significantly at DEPEEP17 and DEPEEP12. There was an HR increase at INPEEP27, INPEEP 32, and DEPEEP17 (*p* < 0.0001; *p* < 0.0001; and *p* < 0.05, respectively), those values being above the normal reference range for the species. The SAP, MAP, DAP, CI, and DO_2_I significantly decreased INPEEP32 (*p* < 0.05).

**Conclusion:**

The ARM by PEEP titration applied in the present study showed better ventilation distribution associated with better aeration in the dependent lung areas, with minimal overdistention between PEEP 17 and 12 cmH_2_O decreasing step. Those changes were also followed by improvements in static and regional compliance associated with increased oxygenation and pulmonary ventilation. ARM promoted a transitory decrease in arterial blood pressure and depression in CI with a concomitant drop in oxygen delivery, which should be best investigated before its routine use in clinical cases.

## Introduction

Healthy horses under general inhaled anesthesia and dorsal recumbency can develop ventilation/perfusion mismatch, probably due to pulmonary atelectasis. Atelectasis can reduce lung functional capacity, decrease lung compliance, reduce the oxygenation ratio, and lead to hypoxemia (1–4).

An alveolar recruitment maneuver (ARM) is a strategy to revert the atelectatic areas. It is based on applying high positive pressure into the lung with the aim of opening collapsed lung units, which can be performed in ways such as PEEP titration or sustained inspiratory pressure. Many previous studies showed that using different kinds of ARM in horses was associated with improved oxygenation and pulmonary mechanics (3, 5).

In humans and small animals, computed tomography (CT) has been used to show the tidal volume migration during the ARM, helping the clinician choose the best ARM and the best PEEP post maneuver, avoiding excessive overdistention and de-recruitment (6, 7).

In large animals such as horses, the CT is not commonly used due to the animal size and the increased risks of radiation exposure to veterinarians and handlers. On the other hand, electrical impedance tomography (EIT) is a relatively new imaging method shown to be an essential tool for ventilation monitoring in horses (8–11). The EIT is a novel technology for humans and small animals capable of showing, in real-time and radiation-free, the ventilation distribution in the lung and the changes in distribution during ventilation and ARM (12, 13). Also, this tool has been widely used in horses to assess pulmonary aeration undergoing anesthesia, mechanical ventilation, recruitment maneuver, standing horses, or for specific procedures (8, 9, 14–16).

However, EIT can identify lung overdistention and atelectasis during mechanical ventilation and show in which region it occurs. Therefore, it is used for monitoring global and regional changes in lung volumes, aeration, ventilation, and heterogeneity. The measurement of just global respiratory mechanics and oxygenation variables to adjust mechanical ventilation may be inappropriate in some situations, as they do not show whether there is hyperdistension, recruitment, or atelectatic areas (12). Araos et al. (16) observed a significant increase in end-expiratory lung volume without a corresponding increase in recruited alveolar volume when using PEEP not preceded by a lung open approach in horses. This fact suggests the pulmonary overdistention of previously aerated alveoli without a significant gain in newly aerated tissue, despite the absence of an increase in alveolar dead space and driving pressure.

Alveolar recruitment maneuvers may increase lung volume and total lung compliance due to inflating previously aerated lung units or recruiting atelectatic alveoli. Although, ARM should specifically recruit atelectatic areas, typically located in the lung regions gravity dependent. Using simple variables such as regional compliance, the center of ventilation, silent space, and tidal distribution index, evaluated by the EIT and associated with respiratory mechanics and oxygenation, this maneuver can be safely titrated (12, 17). Lung areas with impedance changes < 10% of the maximum are silent space areas and can be used as a monitoring tool to guide lung protective ventilation during surgery. Poorly ventilated lung units can mean atelectasis in gravity-dependent lung regions or overinflation in non-dependent regions.

Similarly, the center of ventilation can estimate the air shift between the dorsal and ventral regions of the lungs during mechanical ventilation (18, 19). Changes in compliance of the dependent region may be due to recruited or de-recruited collapsed alveolus, and changes in the non-dependent area may be due to overdistention (10). The tidal distribution index (20) can evaluate the homogeneity distribution during tidal breath considering tidal volume (V_T_) between dependent and non-dependent areas (21).

Although several studies in horses have studied alveolar recruitment maneuvers (2–5), just one study reports the possible occurrence of overinflation (10). Therefore, this study aimed to evaluate regional ventilation distribution during ARM based on lung monitoring assessment by electrical impedance tomography.

We hypothesized that during the descendent PEEP titration, there would be a decrease in areas of pulmonary atelectasis in the dependent regions without promoting overinflation in non-dependent areas assessed by EIT.

## Materials and methods

### Animals

Fourteen Arabian horses with a mean age of 3.0 years and weighing 306 ± 21 Kg, owned by the Department of Surgery—School of Veterinary Medicine and Animal Science of the University of São Paulo, were included in the study. Only healthy horses (ASA physical status I) were used based on medical history, pre-anesthetic physical evaluation, and blood tests (hematology and serum biochemistry). The Ethics Committee on Animal Use of the University of São Paulo, Brazil (No. 7111170715) approved the study.

### Anesthesia and monitoring

Food was withheld for 12 h before anesthesia, and water was given *ad libitum*. On the day of the anesthesia, the right jugular vein was catheterized for drugs and fluid administration, and two 7F introducers (Intro-Flex Percutaneous Sheath Introducers 7F—Edwards Lifesciences, CA, USA.) were placed at the left jugular vein for later hemodynamics measurement (Swan-Ganz Thermodilution Catheters 7F—Edwards Lifesciences, CA, USA, and Central Venous Catheter-Venoseld-−14G 70 cm–VetMedical, Brazil).

The horses were premedicated with detomidine (10 mcg Kg^−1^; IV) and 10 min later induced with ketamine (2.2 mg kg^−1^; IV) and diazepam (0.05 mg kg^−1^; IV). They were intubated with a 24F orotracheal tube and placed in dorsal recumbency on a padded table. The horse was connected to a large animal rebreathing circuit with a microprocessor-controlled anesthesia ventilator (Linea C; Intermed, SP, Brazil), associated with a calibrated differential pressure flowmeter of fixed area type, with a minimum internal diameter of 29 mm. The anesthesia was maintained with isoflurane in 70% oxygen mixed with medical air at an end-tidal concentration (FE'Iso) of 1.5%. Volume-controlled mechanical ventilation was started, with the V_T_ set to 14 ml kg^−1^, the PEEP set to 7 cmH_2_O (baseline ventilation set), and the respiratory rate (*f*_R_) set to maintain the P_E_'CO_2_ at 35–45 mmHg (4.7–6.0 kPa). FE'Iso, FiO_2_, and P_E_'CO_2_ were monitored by a side stream non-dispersive infrared gas analyzer (POET IQ; Criticare System, Inc., WI, USA). The equipment was calibrated before every experiment with a known standard gas mixture containing 1% isoflurane, 5% CO_2_, and 60% nitrous oxide balanced in nitrogen (Criticare Systems, Inc.). The volume sensor was also calibrated before each experiment with a known volume. Offline, the data were analyzed, and total lung static compliance (Cst) was calculated by the formulae Cst = Vt/(Pplateau – PEEP), and oxygen content-based index (F-shunt) was calculated by F-shunt = ([Cc'O_2_ – CaO_2_]/[Cc'O_2_ – CaO_2_] + 3.5 ml dl^−1^) × 100, where 3.5 ml dl^−1^ is a fixed value of C(a-v)O_2_ (22). CI was calculated by the formula CO/BSA; DO_2_I was calculated as follows: CO × CaO_2_ × 10/ BSA, where body surface area (BSA in m^−2^) was calculated using a conversion factor appropriate for horses (k bodyweight^2/3^, where k = 0.1) and PVRI was calculated by the formula ([MPAP-PAOP]/CI × 80) (1). The capillary oxygen content and arterial oxygen content (23) were calculated as follows, where Hb is hemoglobin in arterial blood: Cc'O_2_ = (Hb × 1.34) + (PAO_2_ × 0.0031); CaO_2_ = (Hb × 1.34 × SaO_2_/100) + (PaO_2_ × 0.0031). The alveolar dead space-tidal volume ratio was calculated as (PaCO_2_ – P_E_'CO_2_)/PaCO_2_ (22).

A multiparametric monitor system (DX-2020, Dixtal Biomedical, São Paulo, SP, Brazil) was used for the continuous evaluation of electrocardiography, heart rate (HR), and systolic (SAP), diastolic (DAP), and mean arterial blood pressure (MAP). A 20-gauge catheter was placed in the facial artery for pressure monitoring, and blood collection in a lithium heparinized 3 ml syringes (BD A-Line; BD, UK) for blood gas analysis (ABL 330, Radiometer Medical ApS, Denmark). The pulmonary artery pressure was monitored by the distal extremity of the pulmonary artery catheter placed into the pulmonary artery through the jugular introducer. The correct position of the pulmonary artery catheter was checked by the pressure pattern waveforms. The cardiac output was calculated using three consecutive measures, injecting 40 ml of cold glucose solution (0–5°C) for 15 s through the catheter placed in the right atrium (2). Lactated Ringer's (10 ml kg^−1^ h^−1^; IV) solution was administered throughout the anesthesia, and ephedrine sulfate was administered in a constant rate infusion of 10 μg kg^−1^minute^−1^ (Efedrin; Cristália Produtos Químicos e Farmacêuticos Ltda, Brazil) to maintain the MAP around 60 mmHg, during most of the procedure (3).

### EIT technique and measurements

A 5 cm wide strip was clipped circumferentially around the thorax over the fifth to sixth intercostal space prior to anesthesia induction, and the skin was cleaned with alcohol. Those procedures were performed to achieve the best electrical contact between the animal and the electrodes. After induction, 32 equidistant electrodes disposed of in an elastic EIT belt were placed around the thorax, and ultrasound gel was applied under the belt to ensure the best conductive (14). The animals were positioned in dorsal recumbency on a paddle table, and the belt position was visually checked and corrected if necessary. The belt was connected to the EIT device (EIT Pioneer Set—Swisstom, Switzerland), and data were obtained at 46 frames per second rate (EIT Monitor STEM—Swisstom, Switzerland). Specific horse image reconstruction software was used (iBex—Versão 1.5, Swisstom, Switzerland) to generate EIT images and calculate the EIT parameters center of ventilation (CoV); compliance of the region of interest (ROI compliance); and tidal distribution index (20) for each evaluation time point.

The CoV was determined as a percentage of ventrodorsal (CoV-VD) extension of the lung region. The results were expressed in the percentage of ventilation, where results >50% indicate ventilation mainly in dependent areas and < 50% indicate ventilation mainly in non-dependent areas of the lung. The changes in the CoV mean ventilation migration through the lung areas.

Regional dynamic compliance (Compl_ROI_) for each ROI was calculated by the formulae (Compl_ROI_ = V_T_ × ROI% / driving pressure), where driving pressure is plateau pressure minus PEEP, and tidal distribution index (20) was determined by the ratio between V_T_ distribution in dependent and non-dependent areas (12). This index was used to evaluate the homogeneity distribution during tidal breath, where values closer to 1 mean more homogeneity aeration (21).

Silent space was determined as the area of the lung in which there was less than a 10% difference in the impedance of the lung tissue, calculated based on a reference line. The reference line is perpendicular to the gravity vector, which runs right through the center of ventilation. It is used to sort the pixels of the lowest stretch category into dependent and non-dependent silence spaces. The silent space value above the ventilation horizon was expressed as a percentage of the total number of pixels within the lung contour and called non-dependent silent spaces (NSSs). The value of the silent space below the ventilation horizon was expressed as a percentage of the total number of pixels within the lung contour and is called dependent silent spaces (DSSs). Dependent in this context means located physically below a reference line within the thorax, while non-dependent means above such a reference line within the thorax (19).

### Study design

After 60 min of baseline ventilation, the electrical impedance tomography and cardiopulmonary data were obtained at the baseline (7 cmH_2_O of PEEP). ARM was performed by PEEP titration from 7 to 32 cmH_2_O in 5 cmH_2_O increments at 5 min intervals, keeping a constant V_T_ (14 ml kg^−1^). Then, PEEP was decreased to 17, 12, and 7 cmH_2_O at 5 min intervals. The EIT data were recorded at the increasing PEEP (INPEEP12; INPEEP17; INPEEP22; INPEEP27; and INPEEP32) and the decreasing PEEP (DEPEEP17; DEPEEP12; and DEPEEP7). Cardiopulmonary and ventilation parameters, as well as arterial blood samples (HR; SAP, MAP, DAP; PaO2/FiO2 ratio; PaCO2; fR; VT; VD/VT; Cst; driving pressure), were obtained at each time point, with the exception of CI, which was collected only at baseline, INPEEP32, and DEPEEP7.

### Statistical analysis

Continuous variables were tested for normality using the Shapiro–Wilk test and were reported as mean ± standard deviation (SD). A time influence was analyzed with a two-factory RM-ANOVA followed by a *post-hoc* Dunnett's test to identify differences between time points and baseline. Statistical significance was attributed when *p* < 0.05. Analyses were performed using GraphPad Prism 6 (GraphPad Software, Inc., CA, USA). Sample size calculations (http://powerandsamplesize.com) demonstrate that five animals would be enough to show changes in the dorsoventral center of ventilation (COV). The data used were a COV minimum difference of 4 and a standard deviation of 2.5%, α = 0.05, and β = 0.2. The values used were obtained from previous studies using electrical impedance tomography in horses (10, 15).

## Results

All the cardiopulmonary and EIT data were normally distributed. There were no significant differences between age and body weight.

**Figure 1:**
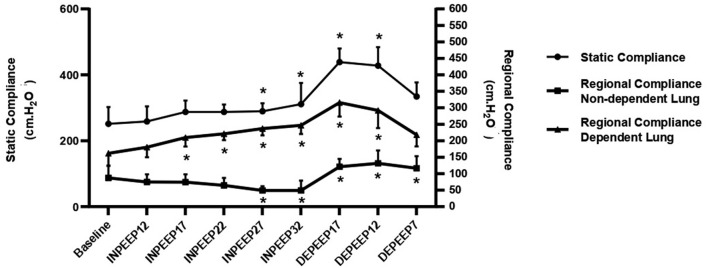
Variation of the static lung compliance (closed circle), regional compliance of non-dependent lung (closed triangle), and regional compliance of dependent lung (closed square) represented by mean ± standard deviation during recruitment maneuver for PEEP titration. PEEP: positive end-expiratory pressure; Baseline: 7 cmH_2_O of PEEP; INPEEP12: PEEP 12 cmH_2_O increase; INPEEP17: PEEP 17 cmH_2_O increase; INPEEP22: PEEP 22 cmH_2_O increase; INPEEP27: PEEP 27 cmH_2_O; INPEEP32: PEEP 32 cmH_2_O increase; DEPEEP17: PEEP 17 cmH_2_O decrease; DEPEEP12: PEEP 12 cmH_2_O decrease; DEPEEP7: PEEP 7 cmH_2_O decrease. ^*^ Statistically significantly different from the baseline.

**Figure 2:**
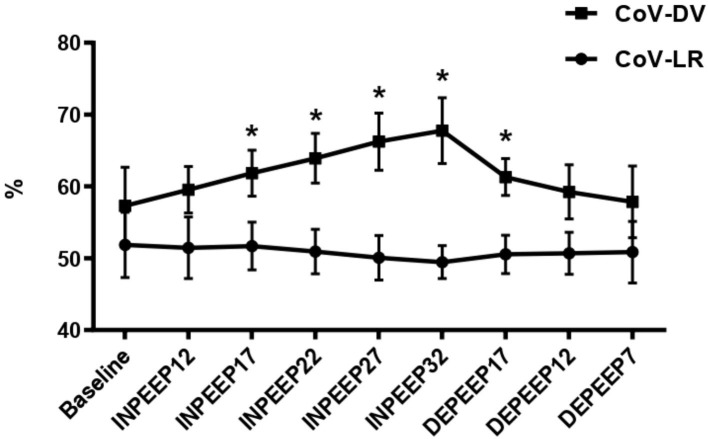
Center of ventilation dorsoventral (closed square) and center of ventilation left–right (closed circle) represented by mean ± standard deviation during recruitment maneuver for PEEP titration. PEEP: positive end-expiratory pressure; Baseline: 7 cmH_2_O of PEEP; INPEEP12: PEEP 12 cmH_2_O increase; INPEEP17: PEEP 17 cmH_2_O increase; INPEEP22: PEEP 22 cmH_2_O increase; INPEEP27: PEEP 27 cmH_2_O; INPEEP32: PEEP 32 cmH_2_O increase; DEPEEP17: PEEP 17 cmH_2_O decrease; DEPEEP12: PEEP 12 cmH_2_O decrease; DEPEEP7: PEEP 7 cmH_2_O decrease. ^*^ Statistically significantly different from the baseline.

**Figure 3:**
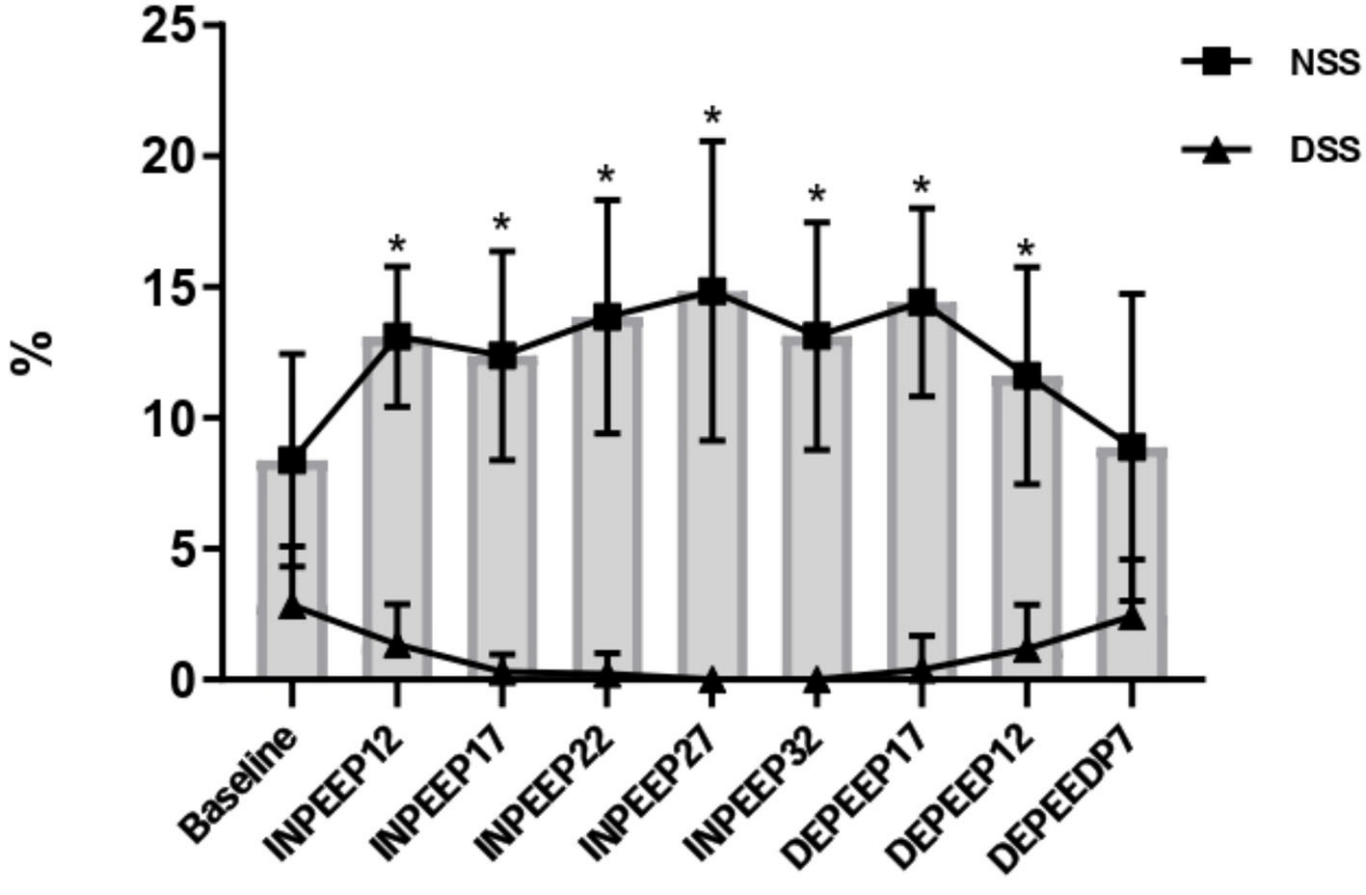
Variation of the non-dependent (closed square) and dependent (closed triangle) silent space region of lung represented by mean ± standard deviation during recruitment maneuver for PEEP titration. NSS: non-dependent silent space; DSS: dependent silent space. PEEP: positive end-expiratory pressure; Baseline: 7 cmH_2_O of PEEP; INPEEP12: PEEP 12 cmH_2_O increase; INPEEP17: PEEP 17 cmH_2_O increase; INPEEP22: PEEP 22 cmH_2_O increase; INPEEP27: PEEP 27 cmH_2_O; INPEEP32: PEEP 32 cmH_2_O increase; DEPEEP17: PEEP 17 cmH_2_O decrease; DEPEEP12: PEEP 12 cmH_2_O decrease; DEPEEP7: PEEP 7 cmH_2_O decrease. ^*^ Statistically significantly different from the baseline.

Data in detail of the HR, SAP, MAP, DAP, CI, SPAP, MPAP, DPAP, PVRI, DO_2_I, PaCO_2_, F-shunt, Cst, PaO_2_, PaO_2_/FiO_2_, driving pressure, V_D_/V_T_, Compl_ROI_, and TDI are shown in Tables 1, 2, and Figure 1. CoV-DV, NSS, and DSS are shown in Figures 2, 3.

**Table 1:** Lung mechanics and electrical impedance tomography data.

**Variable**	**Baseline**	**INPEEP12**	**INPEEP17**	**INPEEP22**	**INPEEP27**	**INPEEP32**	**DEPEEP17**	**DEPEEP12**	**DEPEEP7**
Cst (ml.cmH_2_0^−1^)	252 ± 51	258 ± 46	287 ± 34	287 ± 22	289 ± 24	311 ± 64^*^	438 ± 42^*^	428 ± 56^*^	334 ± 43^*^
Driving Pressure (cmH_2_0)	18 ± 4	18 ± 3	17 ± 3	16 ± 2	16 ± 2	15 ± 2^*^	11 ± 2^*^	11 ± 2^*^	14 ± 2^*^
V_D_/V_T_ (%)	31 ± 5	31 ± 4	31 ± 5	31 ± 4	29 ± 5	28 ± 6	25 ± 4^*^	26 ± 4^*^	28 ± 3
CoV D-V (%)	57 ± 5	59 ± 3	62 ± 3^*^	64 ± 3^*^	66 ± 4^*^	68 ± 5^*^	61 ± 3^*^	59 ± 4	58 ± 5
NSS (%)	8 ± 4	13± 3^*^	12 ± 4^*^	14 ± 4^*^	15 ± 6^*^	13 ± 4^*^	14 ± 4^*^	12 ± 4^*^	9 ± 6
DSS (%)	2.9 ± 2.3	1.3 ± 1.5	0.3 ± 0.7	0.2 ± 0.8	0 ± 0	0 ± 0	0.4 ± 1.3	1.2 ± 1.7	2.4 ± 2.2
Compl_ROI_ Non-dependent (ml cmH_2_0^−1^)	87 ± 37	75 ± 23	74 ± 24	65 ± 22	49 ± 13^*^	49 ± 30^*^	121 ± 24^*^	132 ± 39^*^	116 ± 37
Compl_ROI_ Dependent (ml cmH_2_0^−1^)	162 ± 37	181 ± 30	210 ± 27^*^	221 ± 19^*^	237 ± 21^*^	247 ± 27^*^	315 ± 42^*^	292 ± 54^*^	218 ± 35^*^
TDI	5.7 ± 2.7	4.2 ± 1.1	3.6 ± 1.3^*^	3 ± 1.2^*^	2.1 ± 0.5^*^	2.1 ± 1.5^*^	3.9 ± 0.9^*^	4.7 ± 1.8	5.5 ± 2

Data are expressed as mean ± SD.

* Statistically significantly different from baseline. PEEP,positive end-expiratory pressure; Baseline,7 cmH_2_O of PEEP; INPEEP12,PEEP 12 cmH_2_O increase; INPEEP17,PEEP 17 cmH_2_O increase; INPEEP22,PEEP 22 cmH_2_O increase; INPEEP27,PEEP 27 cmH_2_O; INPEEP32,PEEP 32 cmH_2_O increase; DEPEEP17,PEEP 17 cmH_2_O decrease; DEPEEP12,PEEP 12 cmH_2_O decrease; DEPEEP7, PEEP 7 cmH_2_O decrease; Cst, total pulmonary static compliance; V_D_/V_T_, alveolar dead space-tidal volume ratio; CoVD-V, center of ventilation dorsoventral non-dependent; NSS, non-dependent silent space; DSS, dependent silent space; Compl_Roi_ non-dependent, compliance of the region of interest non-dependent; Compl_Roi_ dependent, compliance of the region of interest dependent; TDI, tidal distribution index.

**Table 2:** Gas exchange and hemodynamics data.

**Variables**	**Baseline**	**INPEEP12**	**INPEEP17**	**INPEEP22**	**INPEEP27**	**INPEEP32**	**DEPEEP17**	**DEPEEP12**	**DEPEEP7**
HR (bpm)	46 ± 11	52 ± 14	56 ± 16	61 ± 17	78 ± 23^*^	85 ± 23^*^	65 ± 18^*^	57 ± 15	55 ± 15
SAP (mmHg)	113.5 ± 11.5	111.1 ± 12.9	106.3 ± 15.7	99.1 ± 26.5	98.3 ± 21.1	82.2 ± 25.8^*^	110.1 ± 27.6	115 ± 22.9	119.1 ± 17.7
MAP (mmHg)	82 ± 12	78 ± 10	75 ± 8	70 ± 16	71 ± 11	62 ± 15^*^	82 ± 20	85 ± 17	88 ± 15
DAP (mmHg)	63.2 ± 13	60.2 ± 11.2	58.9 ± 8.2	55.1 ± 14.3	57.3 ± 11.2	49.6 ± 12.4^*^	66 ± 17.9	68.1 ± 16.1	70.1 ± 15.6
CI (L min^−1^ m^−2^)	2.66 ± 0.54	–	–	–	–	1.29 ±0.74^*^	–	–	2.12 ±0.71
SPAP (mmHg)	20.6 ± 4.5	24.5 ± 8.3	23.8 ± 5.9	24.3 ± 6.6	26.1 ± 6.7^*^	26.1 ± 6.5^*^	20.7 ± 7.5	19.9 ± 7.6	20.3 ± 6.8
MPAP (mmHg)	13.9 ± 2.4	14.9 ± 2.8	16.4 ± 3.2	16.4 ± 4.4	19 ± 5.2^*^	21.1 ± 4.7^*^	13.9 ± 4.5	13.3 ± 3.1	12.7 ± 2.7
DPAP (mmHg)	6.9 ± 4	7.0 ± 4.4	8.3 ± 4.7	9.1 ± 6.7	12.4 ± 6.6^*^	14.8 ± 5.9^*^	5.4 ± 3	6.1 ± 4.1	4.2 ± 3.7
PVRI (dynes s cm^−5^ m^−2^)	243.3 ± 161					442.8 ± 303^*^			289.4 ± 6
DO_2_I (ml min^−1.^m^2^)	530.2 ± 141					363.5 ± 276			415.0 ± 225
PaO_2_ (mmHg) (kPa)	191 ± 71 (25 ± 9)	184 ± 65 (25 ± 9)	190 ± 66 (25 ± 9)	200 ± 66 (27 ± 9)	246 ± 75 (33 ± 10)	286 ± 60 (38 ± 8)	326 ± 51^*^ (43 ± 7)	303 ± 60 (40 ± 9)	273 ± 69^*^ (36 ± 9)
PaO_2_/FiO_2_	260 ± 90	255 ± 89	266 ± 92	282 ± 94	349 ± 106	406 ± 85^*^	451 ± 72^*^	417 ± 92^*^	376 ± 97^*^
PaCO_2_ (mmHg) (kPa)	54 ± 4 (7.3 ± 0.5)	55 ± 4 (7.3 ± 0.5)	54 ± 5 (7.3 ± 0.6)	55 ± 6 (7.4 ± 0.8)	56 ± 6 (7.5 ± 0.8)	56 ± 6 (7.5 ± 0.9)	53 ± 5 (7.1 ± 0.7)	52 ± 6 (6.9 ± 0.8)	51 ± 5 (6.8 ± 0.6)
F-Shunt (%)	16 ± 3	16 ± 3	16 ± 3	15 ± 3	12 ± 4^*^	10 ± 4^*^	8 ± 3^*^	10 ± 4^*^	13 ± 6

Data are expressed as mean ± SD;

* Statistically significantly different from baseline. PEEP, positive end-expiratory pressure; Baseline, 7 cmH_2_O of PEEP; INPEEP12, PEEP 12 cmH_2_O increase; INPEEP17, PEEP 17 cmH_2_O increase; INPEEP22, PEEP 22 cmH_2_O increase; INPEEP27, PEEP 27 cmH_2_O; INPEEP32, PEEP 32 cmH_2_O increase; DEPEEP17, PEEP 17 cmH_2_O decrease; DEPEEP12, PEEP 12 cmH_2_O decrease; DEPEEP7, PEEP 7 cmH_2_O decrease; HR, heart rate; SAP, systolic arterial pressure; MAP, mean arterial pressure; DAP, diastolic arterial pressure; IC, cardiac index; PVRI, pulmonary vascular resistance index; DO_2_I, oxygen delivery index; PaO_2_, arterial partial pressure of oxygen; PaO_2_/FiO_2_, ratio of arterial partial pressure of oxygen and oxygen inspiratory fraction; F-shunt, oxygen content-based index.

### Effects of RM on EIT data

The CoV-DV increased significantly in the moments INPEEP17 (*p* < 0.05), INPEEP22 (*p* < 0.0001), INPEEP27 (*p* < 0.0001), INPEEP32 (*p* < 0.0001), and DEPEEP17 (*p* < 0.05) when compared to baseline.

Associated with the CoV-DV increase, there was a significant decrease in the DSS during the ARM compared to the baseline. The NSS was significantly increased when PEEP was applied above 12 cmH_2_O compared to baseline.

The Compl_ROI_ showed a significant increase in the dependent area at INPEEP17 (*p* = 0.0019); INPEEP22 (*p* < 0.0001); INPEEP27 237 (*p* < 0.0001); INPEEP32 (*p* < 0.0001); DEPEEP17 (*p* < 0.0001); DEPEEP12 (*p* < 0.0001); DEPEEP7 (*p* = 0.0002) and a significant decrease in the non-dependent area [INPEEP27 (*p* = 0.0229); INPEEP32 (*p* = 0.0224); DEPEEP12 (*p* = 0.0047)], both compared to their baseline values.

Associated with the CoV-DV, silence space, and Compl_ROI_, there was a significant decrease in the TDI in the moments at INPEEP17 (*p* = 0.0073); INPEEP22 (*p* = 0.0002); INPEEP27 (*p* < 0.0001); INPEEP32 (*p* < 0.0001); DEPEEP17 (*p* = 0.0320) when compared to the baseline.

### Effects of RM on lung mechanics and oxygenation data

The drive pressure decreased significantly during the ARM in INPEEP32 (*p* < 0.01), DEPEEP17 (*p* < 0.0001), DEPEEP12 (*p* < 0.0001), and DEPEEP7 (*p* < 0.0001) compared to baseline results. The Cst, PaO_2_, and PaO_2_/FiO_2_ ratio increased significantly from INPEEP32 until DEPEEP7 (*p* < 0.05). The V_D_/V_T_ decreased significantly at DEPEEP17 and DEPEEP12 compared to baseline.

### Effects of RM on hemodynamics data

Heart rate increased at INPEEP27, INPEEP 32, and DEPEEP17 compared to baseline (*p* < 0.0001; *p* < 0.0001; and *p* < 0.05, respectively), with those values being above the normal for the species. The SAP, MAP, and DAP had a significant decrease at INPEEP32 compared to baseline (*p* < 0.05), which was also observed in relation to CI (*p* < 0.01), SPAP, MPAP, DPAP (*p* < 0.05), and PVRI (*p* < 0.05), but both variables returned to baseline values at DEPEEP7. There was a significant decrease in F-shunt at INPEEP2, INPEEP32, DEPEEP17 and DEPEEP12 compared to baseline (*p* < 0.05; *p* < 0.001; *p* < 0.0001; *p* < 0.001, respectively).

## Discussion

The alveolar recruitment maneuver by PEEP titration applied in the present study showed changes in pulmonary aeration assessed by EIT, mechanical ventilation, and oxygenation. The best total lung compliance associated with a lower driving pressure and alveolar dead space-tidal volume ratio occurred between PEEP 17 and PEEP 12 cmH_2_O in the decreased step of titration. Moreover, the best oxygenation showed by PaO_2_/FiO_2_ was also seen in this step. Concerning the regional assessment by EIT, there was better ventilation distribution associated with the aeration shift to dependent lung areas, with minimal overdistention compared to that observed before ARM. The optimal PEEP is found in the descending branch of the titration and can be identified by monitoring the decrease in PaO_2_ and lung compliance (24, 25). The optimal post-ARM PEEP in humans corresponds to 2 cmH_2_O higher than the nearest PEEP close to the alveolar collapse. However, overdistended regions previously opened would be mascaraed when just global variables are assessed. Therefore, the EIT data can individualize dependent and non-dependent pulmonary regions and correctly guide recruitment to prevent overdistention (10, 12).

Ambrósio et al. (2) observed that the best PEEP value for the best static compliance was between 20, 15, and 10 cmH_2_O of the descending titration step. Ambrisko et al. (10) also observed the best static compliance during the descending titration phase between PEEP 20, 15, and 10 cmH_2_O. Values found in our actual study are similar because optimal PEEP values for the best compliance were between DEPEEP17 and DEPEEP12. Andrade et al. (3) also found the optimal PEEP for the best static compliance between 17 and 12 cmH_2_O of the titration descending step. Furthermore, in the four cited articles, the best compliance values for the optimal PEEP from 20 to 10 cmH_2_O of the descending titration step were also accompanied by better oxygenation values and lower alveolar dead space-tidal volume ratio.

The regional compliance of the dependent area assessed by EIT increased during ARM and presented the best values at PEEP 17 and 12 cmH_2_O of the descending titration step. However, Ambrisko et al. (10) observed an increase in the regional compliance of the dependent lung in PEEP 25 and 20 cmH_2_O of the descending titration step. This difference was probably due to the difference in the recruitment maneuver itself. In the Ambrisko et al. (10) study, the maximal peak inspiratory pressure and driving pressure was higher than in the present study. Furthermore, in the present study, both static compliance and regional dependent compliance were maintained significantly elevated until PEEP 7 cmH_2_O decreasing titration step. On the other hand, Ambrisko et al. (10) observed that static compliance and regional compliance gradually decreased as PEEP decreased from 10, 5, and 0 cmH_2_O, demonstrating a de-recruitment of the dependent lung areas.

Pulmonary ventilation moved toward the dependent areas and was confirmed by the shift of the CoV-DV from INPEEP17 to DEPEEP17, following the ARM and returned to baseline values from DEPEEP12 as also observed by Ambrisko et al. (10). These data clearly show the effect of ARM and subsequent de-recruitment occurring when PEEP was decreased from 12 to 7 cmH_2_O, in agreement with the open lung approach previously reported by Lachman (26).

Furthermore, the present study showed a decrease in the TDI, indicating a better distribution in the tidal volume at INPEEP 17. However, values closer to 1 were observed at INPEEP 27 and INPEEP 32 during the highest steps of ARM. TDI is an important index used to evaluate the aeration distribution ratio between the dependent and non-dependent lung areas. It is an indirect homogeneity parameter where values closer to 1 mean more homogeneity aeration (21). Although, in the present study, more homogenous lung aeration did not coincide with the optimal PEEP for oxygenation and CoV-DV. Otherwise, Spadaro et al. (21) demonstrated in human beings that incremental PEEP levels resulted in a significant reduction of TDI at PEEP 10 and a 5 cmH2O decreasing step. Ambrósio et al. (12) observed in dogs submitted to recruitment maneuvers assessed by IET that TDI and CoV-DV variables could not detect significant changes. However, these variables represent a direction toward ventilation from non-dependent to dependent regions of the lungs.

Another EIT data evaluated in this present study were the NSS, which showed a significant increase at the beginning of ARM, increasing significantly from 8% at baseline to 15% at PEEP 27 cmH2O. These results associated with the decrease in the non-dependent regional compliance strongly suggest overdistention with the highest values of PEEP. In the present study, the lowest value of NSS was associated with the highest values of non-dependent regional compliance in DEPEEP12. Therefore, based on the optimal PEEP, where there is less overdistention and the best recruitment of the dependent areas, DEPEEP12 seems to be the best value. Although DSS did not present significant values, it showed a total decrease (0%) in the highest PEEP in the ARM, but the lowest values were still observed in DEPEEP17 and DEPEEP12 compared to the baseline. According to these data, the best dependent regional compliance occurred in DEPEEP17 and DEPEEP12. In dogs, Ambrósio et al. (12) observed an increase in the NSS which was also shown in the highest values of PEEP during ARM, suggesting possible overinflation of the de non-dependent region of the lung. The optimal PEEP to maintain ARM was localized in the decreasing titration step. Hopster et al. (27) also verified alveolar overdistention after ARM by PEEP titration in horses; however, no signs of ventilator-induced lung injury were confirmed based on histopathological and cytological analyses. Therefore, the optimal PEEP values set after ARM should be the balance between the best regional compliance in the dependent and non-dependent lung areas (23). Ukere et al. (19), in a human study, concluded that silent space measurements seem to be an excellent tool to individualize PEEP in bedsides patients.

The best static compliance, regional dependent compliance, and oxygenation also occurred at DEPEEP 17 and DEPEEP12, associated with the lowest F-shunt. Despite the CI decreasing 53.8 % at INPEEP32 and HR increasing 84% at the same moment, they gradually returned to baseline values in DEPEEP 17 and DEPEEP12. SAP, MAP, and DAP decreased significantly at DEPEEP32 but returned to baseline values with a decrease in PEEP. It is already known that the high intrathoracic and transpulmonary pressure resulting from the mechanical ventilation associated with PEEP may cause temporary hemodynamic instability (1). Wettstein et al. (4) observed that ponies undergoing ARM with PEEP titration presented limited adverse cardiovascular effects. Hopster et al. (5), studying ARM in horses submitted to colic surgery, observed a decrease in MAP during ARM, but no difference in dobutamine requirement was noted. Ambrósio et al. (2) in adult horses observed a decrease in CO at the highest PEEP level during ARM by titration; however, any significant changes occurred in HR and MAP. In the study of Hopster et al. (28) in adult horses submitted to ARM and PEEP in dorsal or lateral recumbency, the authors did not observe differences in MAP, HR, and dobutamine requirements compared to the sham group. Andrade et al. (3) reported mild cardiovascular changes with PEEP 17 and 12 cmH_2_O in the decreasing step after ARM, successfully treated with ephedrine, without changes in MAP. The differences shown by these articles regarding the hemodynamic side effects during ARM can be more pronounced in patients with hypovolemia. However, they can be attenuated with a fluid load before ARM or vasoactive drugs (5, 29, 30). In the present study, there were no differences in ephedrine or fluid load requirement, probably because the animals were not dehydrated, and the decrease in CI was transitory. There was no statistical difference in DO_2_I, but the variable presented a tendency to decrease in the higher value of PEEP due to the decrease in CI at the same time point. This decrease is also expected during the maneuver as the DO_2_I depends mainly on the cardiac output and arterial oxygen content. The increase in PaO_2_ does not contribute to a substantial change in oxygen content values, and consequently, a decrease in DO_2_I is expected. As already mentioned, this decrease is transitory. But it must be reinforced that this kind of maneuver should be performed with caution in animals with severe hemodynamic compromise. Regarding the pulmonary pressure variables, the changes verified are also predictable since the squeezing of the pulmonary vasculature by the alveolar expansion promotes an increase in pulmonary resistance. These changes are also short-lived and extreme in this model of ARM, lasting only 5 min, but they are most ephemeral in clinical practice.

There are some limitations in the present study related to ARM in healthy lungs that can be different in sick lungs, requiring a higher level of PEEP to maintain the lungs open after ARM. In the same situation, horses that presented acute colic underwent ventral mid-line laparotomy can need more pressure to open and keep the lungs open. The lack of cardiac output monitoring to assess cardiovascular function in all ARM moments was another technical limitation in the study methods. A specific EIT limitation could be that EIT images represent a single cross-section of the thorax and lungs around the fifth and sixth intercostal space. That EIT localization did not allow assessing most caudal regions because we used a single-plane EIT, which could show the changes in thoracic structures cranially and caudally to the belt position only. It was not possible to confirm whether the ARM was capable of opening the lungs and keeping them open in other regions at the time. A new study with two-plane EIT to generate a 3D image should be evaluated to ensure the ARM in other lung regions (31).

## Conclusion

In conclusion, the EIT technique was capable of assessment of the changes in regional ventilation distribution during ARM by PEEP titration by shifting the center of ventilation, dependent and non-dependent regional compliance, and tidal distribution index. The EIT associated with oxygenation, respiratory mechanics, and the hemodynamic data suggest the optimal PEEP capable of keeping the alveolus open after ARM, associated with lower overdistention in the non-dependent areas, occurred at PEEP 12 cmH_2_O decreasing titration step. ARM resulted in depression of CI and a transitory decrease in arterial pressure with a concomitant drop in oxygen delivery, which should be investigated in detail before its routine use in clinical cases.

## Data availability statement

The original contributions presented in the study are included in the article/supplementary material, further inquiries can be directed to the corresponding author.

## Ethics statement

The animal study was reviewed and approved by Ethics Committee on Animal Use of the University of São Paulo, Brazil.

## Author contributions

FA participated in study design and experimental procedure, performed the statistics, performed experimental procedures, interpreted the data, and drafted the manuscript. AA designed the study, performed experimental procedures, analyzed the data, and wrote the manuscript. RR, LF, LG, SG, RS, and TR participated in the experimental procedure. MP performed the statistics, performed figures, and interpreted the data. DF designed the study, performed experimental methods, and critically reviewed the manuscript. All authors contributed to the critical revision of the manuscript and approved the final manuscript.
